# Patterns of Treatment and Outcomes in Older Men With Penile Cancer: A SEER Dataset Analysis

**DOI:** 10.3389/fonc.2022.926692

**Published:** 2022-06-29

**Authors:** Maria T. Bourlon, Haydee Verduzco-Aguirre, Elizabeth Molina, Elisabeth Meyer, Elizabeth Kessler, Simon P. Kim, Philippe E. Spiess, Thomas Flaig

**Affiliations:** ^1^ Department of Hemato-Oncology, Instituto Nacional de Ciencias Médicas y Nutrición Salvador Zubirán, Mexico City, Mexico; ^2^ Population Health Shared Resource, University of Colorado Cancer Center, Aurora, CO, United States; ^3^ Division of Medical Oncology, University of Colorado School of Medicine, Aurora, CO, United States; ^4^ Division of Urology, University of Colorado-Denver, Denver, CO, United States; ^5^ Department of Genito-Urinary Oncology, H. Lee Moffitt Cancer Center and Research Institute, Tampa, FL, United States

**Keywords:** penile cancer, older adults, geriatric oncology, SEER program, risk factors

## Abstract

**Purpose:**

To evaluate clinicopathologic and treatment characteristics from a population-based cohort of penile cancer, with an emphasis in older adults, due to incomplete evidence to guide therapy in this age subgroup.

**Materials and Methods:**

Patients with malignant penile tumors diagnosed 2004-2016 were identified in the Surveillance, Epidemiology and End Results Program (SEER)-18 dataset. Demographic and treatment characteristics were obtained. Population was analyzed by age at diagnosis (<65 vs ≥65 years). We examined univariate associations between age groups with Chi-square analysis. To study survival, we calculated Kaplan-Meier survival curves, but due to the high number of competing events, we also performed a univariate competing risk analysis using the cumulative incidence function, and a multivariate analysis using the Fine-Gray method. We also described competing mortality due to penile cancer and other causes of death.

**Results:**

We included 3,784 patients. Median age was 68 years, 58.7% were aged ≥65. Older patients were less likely to have received chemotherapy (p<0.001), primary site surgery (p = 0.002), or therapeutic regional surgery (p <0.001). Median overall survival (OS) in patients <65 years was not reached (95% CI incalculable) vs 49 months in those ≥65 years (95% CI 45-53, p <0.0001). On univariate analysis, age was associated with a lower incidence of penile cancer death. On multivariate analysis, stage at diagnosis, and receipt of primary site surgery were associated with a higher incidence of penile cancer death. Estimated penile cancer-specific mortality was higher in patients <65 years in stages II-IV. Estimated mortality due to other causes was higher in older patients across all stages.

**Conclusions:**

Older patients are less likely to receive surgery, chemotherapy and radiotherapy for penile cancer. Primary surgical resection was associated with better penile cancer-specific mortality on multivariate analysis. Competing mortality risks are highly relevant when considering OS in older adults with penile cancer. Factors associated with undertreatment of older patients with penile cancer need to be studied, in order to develop treatment strategies tailored for this population.

## Introduction

Penile cancer is a rare malignancy, comprising less than 1 percent of male cancers in the US (1). Over 95% of penile cancers have a squamous histology. Human papillomavirus (HPV) is identified in 30 to 50% of cases. Other risk factors include a history of phimosis, human immunodeficiency virus (HIV) infection, tobacco exposure, poor sexual hygiene, multiple sexual partners, and a history of sexually transmitted infections (2).

A key prognostic factor in penile cancer is the lymph node status, with a 5-year OS of 93% in patients without nodal involvement and a median survival of less than a year in those with pelvic node involvement.

There is limited high-quality evidence to guide the treatment of penile cancer. Early-stage disease is generally managed with a limited excision or radiation therapy (3). More advanced disease requires more extensive surgery, such as total penectomy, with some form of regional node treatment according to clinical findings and risk of nodal involvement (4). Chemotherapy is reserved for advanced stages, given as neoadjuvant or palliative treatment (5).

Data regarding outcomes in older adults are limited, despite a median age at diagnosis of 68 years (6). Older adults are under-represented in the few prospective trials in penile cancer (7). The lack of information regarding tolerance to treatment and expected prognosis in this population may make older adults susceptible to both undertreatment and use of futile treatments with an adverse impact in quality of life. Geriatric oncology guidelines provide guidance regarding the screening of older patients for frailty, and using physiologic, rather than chronologic age, to guide treatment decisions (8). Currently, most disease-specific treatment guidelines provide general recommendations for the treatment of penile cancer in older adults based largely on expert opinion (9).

Our aim was to describe differences in characteristics and survival of patients with penile cancer according to age. Our hypothesis was that older adults with penile cancer have a poorer overall survival (OS) and cancer-specific survival (CSS) irrespective of stage.

## Materials and Methods

**Figure 1:**
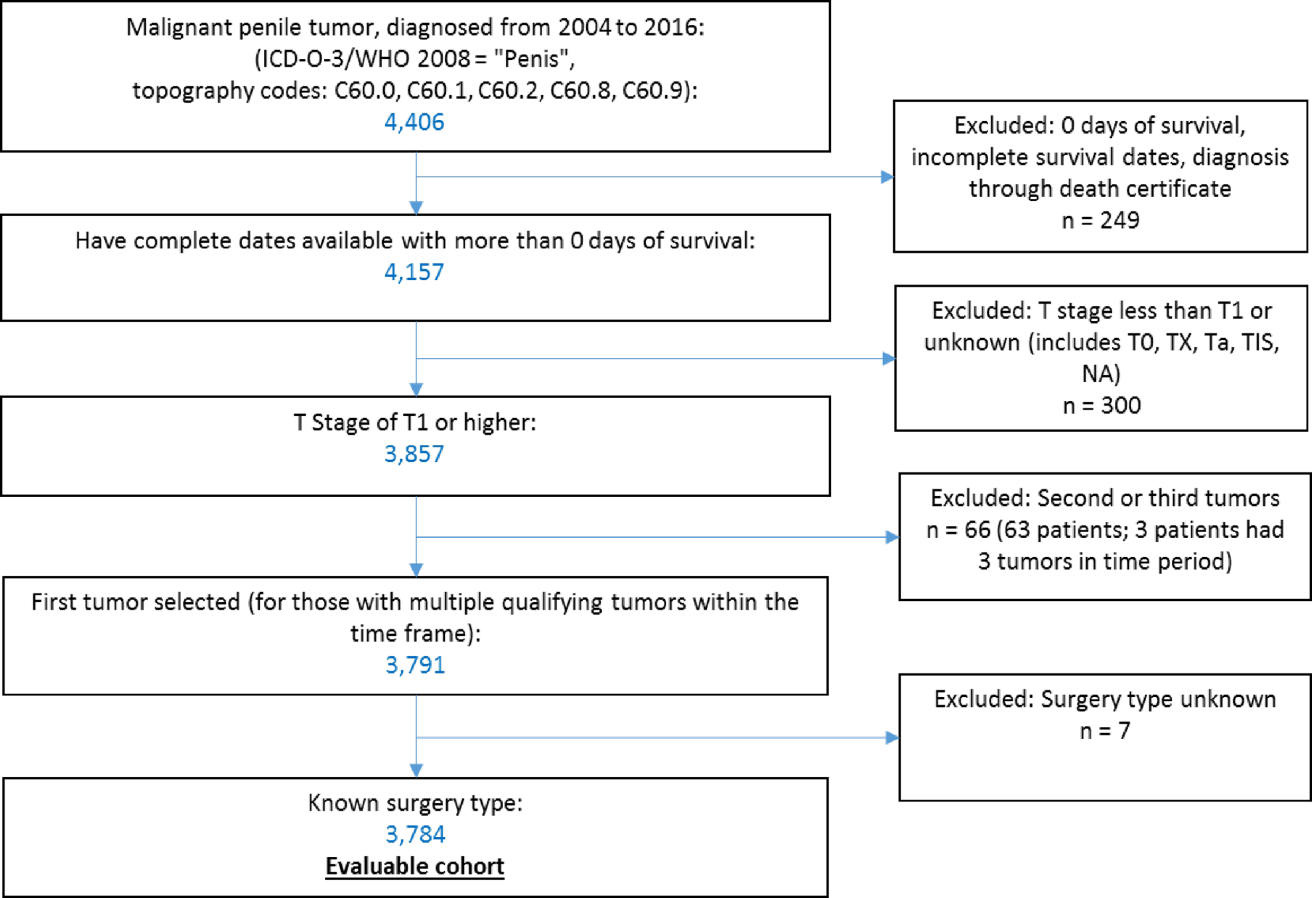
Selection of the study cohort.

Data were accessed through SEER*Stat software (10) to select qualifying cases within the Surveillance, Epidemiology and End Results Program (SEER)-18 dataset, which covers 27.8% of the US population (11). We identified 4,406 patients with malignant penile tumors (ICD-O-3 topography codes C60) diagnosed from 2004 to 2016. We excluded patients with zero days of survival or incomplete survival data (n = 249), with a T stage less than T1 (T0, Tx, Ta, Tis) or unknown (n = 300). For patients with multiple qualifying penile tumors in our period of observation (n = 63), we selected their first tumor within the time frame. We excluded patients with unknown primary surgery type (n = 7). Application of these criteria resulted in 3,784 patients in the final sample (**Figure 1**).

We obtained demographic and treatment characteristics. Stage at diagnosis was described using Derived AJCC 6^th^ edition (DAJCC 6^th^ ed) (for cases diagnosed from 2004 through 2015) or SEER Derived Combined Stage Group (for cases diagnosed in 2016). We obtained information on surgery (both primary and regional), known chemotherapy and known radiation therapy, as well as cause of death. We divided the population according to age at diagnosis (<65 years vs. ≥65 years). Continuous variables are presented with the median and range values, and categorical data are presented as frequencies and proportions.

To study survival, due to the high number of competing events due to death from other causes, we performed a competing risk analysis to evaluate both penile cancer-specific mortality and other cause mortality at 5 years. We performed a univariate analysis using the cumulative incidence function (CIF) to calculate the probability of each event and used Gray’s test to compare CIFs between groups. We then performed a multivariate analysis through the Fine-Gray model using death due to penile cancer as the event of interest, and other causes of death as competing events. We also described the comparison of results from this model with those obtained with Kaplan-Meier survival curves and a Cox regression model. Schoenfeld residuals were examined to test the proportional hazards assumption in the Cox models. Violations were addressed using time-dependent interaction terms.

All statistical analyses were completed using SAS 9.4 (SAS Institute, Cary NC) and evaluated at a significance level of p < 0.05. This study used population-based data only and is therefore not human subjects research requiring IRB approval.

## Results

We included 3,784 patients. Median age of the population was 68 years old; 2,222 (58.7%) patients were older (≥65 years) at diagnosis. Population characteristics are reported in **Table 1**. There was a higher proportion of white (non-Hispanic) (71.0 vs 54.0%) patients in the older group, whereas the proportion of Hispanic patients was higher in younger patients (28.7 vs 13.9%) (p <0.0001). Older patients were more likely to be insured (69.5 vs 48.5%, p <0.0001), with insurance status unknown in about 20% of patients. Patients ≥65 years had a lower proportion of advanced disease (stage III-IV), (21.6% versus 27.3%, p = 0.0007).

**Table 1:** Population characteristics according to age at diagnosis (<65 years vs ≥65 years).

Characteristic	Overall, n (%)	<65 years, n (%)	≥65 years, n (%)	P value
All patients	3784 (100)	1562 (41.3)	2222 (58.7)	
Age (median, inter-quartile range)	68 (58-78)	55 (48-60)	76 (70-82)	<0.0001
Year of diagnosis				0.054
2004-2009	1561 (41.3)	673 (43.1)	888 (40.0)	
2010-2016	2223 (58.7)	889 (56.9)	1334 (60.0)	
Race/ethnicity				<0.0001
White (non-Hispanic)	2416 (63.8)	843 (54.0)	1573 (70.8)	
Black (non-Hispanic)	384 (10.1)	181 (11.6)	203 (9.1)	
Hispanic	757 (20.0)	446 (28.6)	311 (14.0)	
Asian or Pacific Islander	166 (4.4)	65 (4.2)	101 (4.5)	
Other/unknown	61 (1.6)	27 (1.7)	34 (1.5)	
Marital status				<0.001
Married (including common law)	2114 (55.9%)	834 (53.4%)	1280 (57.6%)	
Single (never married)	617 (16.3%)	395 (25.3%)	222 (10.0%)	
Divorced	349 (9.2%)	159 (10.2%)	190 (8.6%)	
Widowed	362 (9.6%)	34 (2.2%)	328 (14.8%)	
Separated	39 (1.0%)	18 (1.2%)	21 (0.9%)	
Unmarried or domestic partner	4 (0.1%)	3 (0.2%)	1 (0.1%)	
Unknown	299 (7.9%)	119 (7.6%)	180 (8.1%)	
Insurance status				<0.0001
Insured	2296 (60.7)	757 (48.5)	1539 (69.3)	
Medicaid	484 (12.8)	291 (18.6)	193 (8.7)	
Uninsured	160 (4.2)	154 (9.9)	6 (0.3)	
Unknown	160 (4.2)	65 (4.2)	95 (4.3)	
Prior to 2007 (not captured)	684 (18.1)	295 (18.9)	389 (17.5)	
Stage at diagnosis^a^				0.0007
I	1923 (50.8)	773 (49.5)	1150 (51.8)	
II	783 (20.7)	296 (19.0)	487 (21.9)	
III	578 (15.3)	263 (16.8)	315 (14.2)	
IV	330 (8.7)	164 (10.5)	166 (7.5)	
Unknown	170 (4.5)	66 (4.2)	104 (4.7)	
Histology				
Squamous cell carcinoma	3600 (95.1%)	1504 (96.3%)	2096 (94.3%)	0.005
Other	184 (4.9%)	58 (3.7%)	126 (5.7%)	

a Stage using Derived AJCC 6^th^ edition (DAJCC 6^th^ ed) (for cases diagnosed from 2004 through 2015) or SEER Derived Combined Stage Group (for cases diagnosed in 2016).

After a median follow-up time of 60 months, 2131 patients were alive. 599 died from penile cancer, and 1054 from other competing causes. Using the Kaplan-Meier method, five-year OS was 54.6%, and five-year CSS was 79.8%. Median OS in patients <65 years was not reached (95% CI incalculable) vs 49 months in those ≥65 years (95% CI 45-53, p <0.0001).

**Figure 2:**
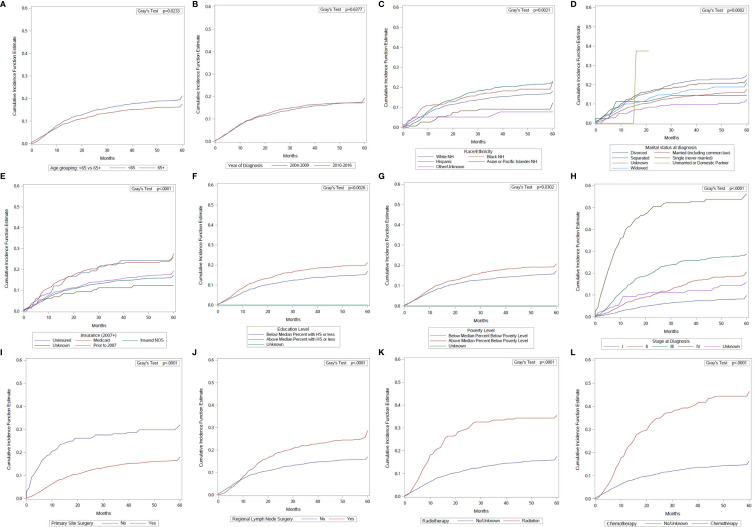
Cumulative incidence function curves of penile cancer mortality according to: **(A)** age at diagnosis; **(B)** year of diagnosis; **(C)** race/ethnicity; **(D)** marital status; **(E)** insurance status; **(F)** education level; **(G)** poverty level; **(H)** stage at diagnosis; **(I)** primary site surgery; **(J)** regional surgery; **(K)** radiotherapy use; **(L)** chemotherapy use.

Univariate analysis using the cumulative incidence function showed that age at diagnosis, year of diagnosis, race/ethnicity, marital status, insurance status, education level, poverty level, stage at diagnosis, primary site surgery, regional nodal surgery, radiotherapy, and chemotherapy, were significantly associated with death from penile cancer (**Figure 2**). On univariate analysis, age was associated with a lower incidence of penile cancer death.

**Figure 3:**
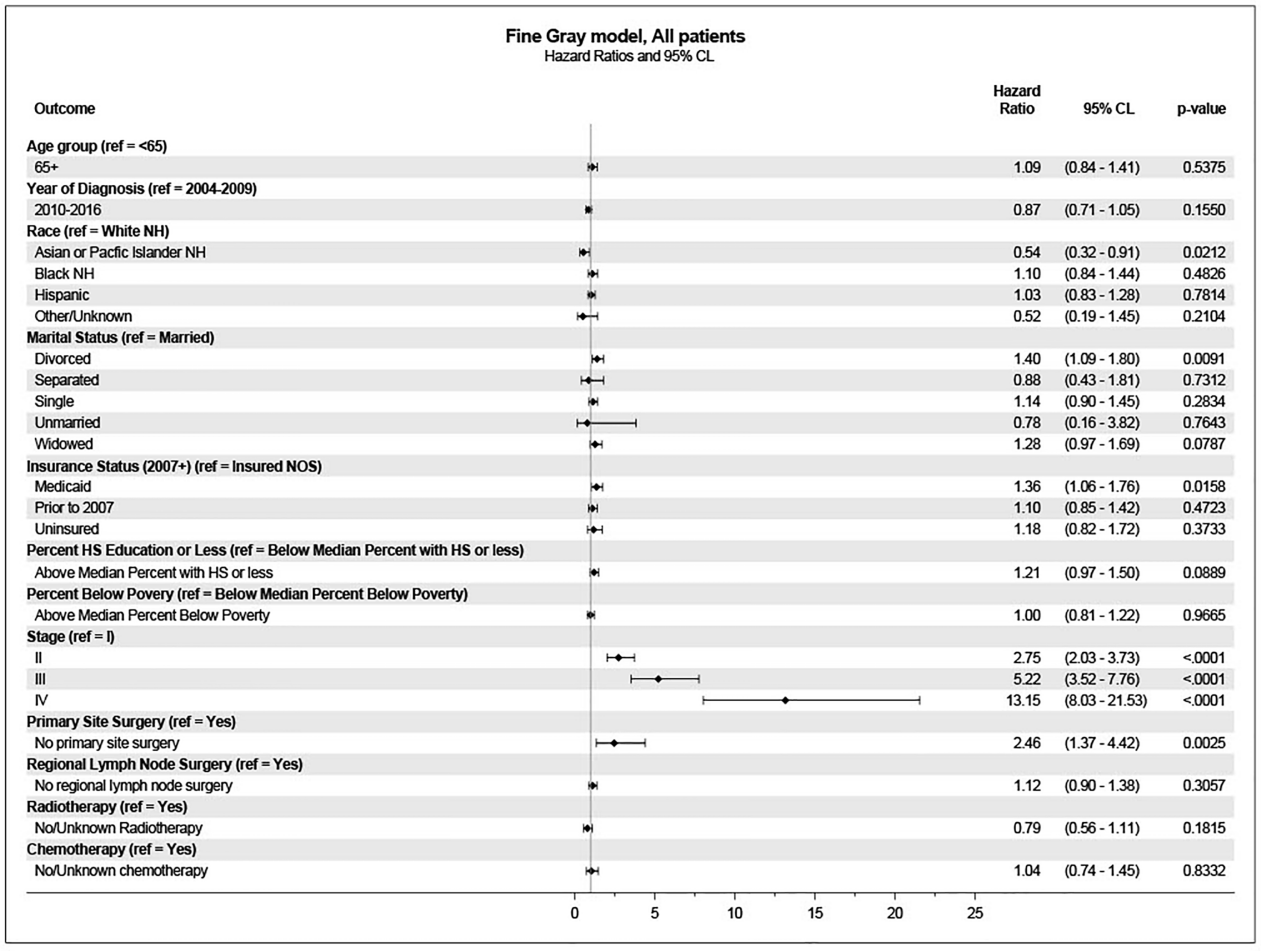
Multivariate analysis for penile cancer survival (Fine-Gray model).

These variables were included in the multivariate analysis for survival using the Fine-Gray model. In this analysis, age was not associated with a higher incidence of penile cancer death. Significantly associated variables included stage at diagnosis, primary site surgery, marital status, insurance status, and race/ethnicity (**Figure 3**).

We incorporated mortality due to causes other than penile cancer into the cumulative incidence curves, stratifying the population by age and stage at diagnosis. These data are presented in **Table 2**. Estimated penile cancer-specific mortality varied from 8.5% to 64.4% for younger patients, and from 10.7% to 48.4% for older patients. Estimated mortality due to other causes was higher in older patients across all stages. Causes of death by age are shown in **Table 3**.

**Table 2:** 5-year competing mortality stratified by age at diagnosis and stage at diagnosis.

		Stage I	Stage II	Stage III	Stage IV
<65	Penile cancer	8.5% (6.5-10.9)	23.8% (18.0-30.0)	31.8% (25.6-38.2)	64.4% (55.1-72.3)
	Other neoplasms	4.7% (3.1-6.8)	2.9% (1.1-6.4)	2.5% (0.9-5.5)	2.0% (0.5-5.3)
	Cardio/cerebro vascular	5.6% (3.9-7.7)	2.6% (1.1-5.3)	4.7% (2.4-8.3)	3.5% (1.1-8.3)
	Other causes^a^	8.3% (6.2-10.8)	8.4% (4.8-13.3)	14.6% (9.8-20.4)	5.9% (2.6-10.9)
≥65	Penile cancer	10.7% (8.8-12.9)	18.7% (14.8-23.0)	26.3% (21.3-31.7)	48.4% (40.1-56.2)
	Other neoplasms	10.2% (8.3-12.3)	8.9% (6.1-12.3)	12.5% (8.7-17.0)	7.4% (3.9-12.4)
	Cardio/cerebro vascular	19.4% (16.7-22.2)	14.7% (10.9-18.9)	12.1% (8.2-16.7)	8.9% (4.4-15.5)
	Other causes^a^	23.6% (20.7-26.5)	30.8% (25.7-35.9)	22.7% (17.7-28.2)	23.3% (16.5-30.8)

a Other causes include: chronic liver disease and cirrhosis, chronic obstructive pulmonary disease and allied conditions, diabetes mellitus, infections, Alzheimer’s disease, renal disease, accidents, suicide and homicide, and those coded as other causes or not specified.

**Table 3:** Cause of death by age group.

Cause of death (n, %)	Overall population N=3784	Age at diagnosis <65 N=1562	Age at diagnosis ≥65 N=2222
Penile cancer	599 (15.8%)	270 (17.3%)	329 (14.8%)
Other neoplasms	210 (5.5%)	41 (2.6%)	169 (7.6%)
Cardiovascular and cerebrovascular	316 (8.3%)	54 (3.5%)	262 (11.8%)
Other causes^a^	528 (13.9%)	102 (6.5%)	426 (19.2%)
Censored	2131 (56.3%)	1095 (70.1%)	1036 (46.6%)

a Other causes include: chronic liver disease and cirrhosis, chronic obstructive pulmonary disease and allied conditions, diabetes mellitus, infections, Alzheimer’s disease, renal disease, accidents, suicide and homicide, and those coded as other causes or not specified.

We also investigated received treatments by age. Older patients were less likely to have received chemotherapy in the overall population (6.7% vs 14.9%, p <0.0001), as well as at each stage at diagnosis. There was no difference in use of radiotherapy according to age (7.9% vs 9.3% p = 0.11) in the whole population; however, older patients were less likely to have received radiotherapy with stages III and IV, but more likely to have received it when diagnosed at stage I. Older patients were less likely to have any primary site surgery (91.4% vs. 94.0%, p=0.002); this was also statistically significant for stages I (91.5% vs 95.5%, p=0.0007) and IV (70.5% vs 81.1%, p=0.024). Among the 3478 patients with information on type of primary site surgery, there was no difference between the receipt of radical and partial primary site surgery according to age: 13.7% of younger patients underwent radical surgery, in comparison to 15.7% of older patients (p = 0.419). Patients aged ≥65 were also less likely to have therapeutic regional surgery (13.3% vs 24.5%, p <0.001). (**Table 4**).

**Table 4:** Treatment modalities by age and stage at diagnosis.

		Total (n, %)	<65 years (n, %)	≥65 years (n, %)	P value
Chemotherapy	All stages	380/3784 (10.0%)	232/1562 (14.9%)	148/2222 (6.7%)	<0.001
	Stage I	53/1923 (2.8%)	30/773 (3.9%)	23/1150 (2.0%)	0.01
	Stage II	55/783 (7.0%)	36/296 (12.2%)	19/487 (3.9%)	<0.001
	Stage III	120/578 (20.8%)	77/263 (29.3%)	43/315 (13.7%)	<0.001
	Stage IV	150/330 (45.5%)	88/164 (53.7%)	62/166 (37.4%)	0.002
	Stage unknown	2/170 (1.18%)	1/66 (1.5%)	1/104 (1.0%)	0.74
Radiotherapy	All stages	321/3784 (8.5%)	146/1562 (9.3%)	175/2222 (7.9%)	0.10
	Stage I	74/1923 (3.8%)	21/773 (2.7%)	53/1150 (4.6%)	0.03
	Stage II	58/783 (7.4%)	24/296 (8.1%)	34/487 (7.0%)	0.55
	Stage III	88/578 (15.2)	41/263 (15.6%)	47/315 (14.9%)	0.049
	Stage IV	98/330 (29.7%)	59/164 (36.0%)	39/166 (23.5%)	0.01
	Stage unknown	3/170 (1.8%)	1/66 (1.5%)	2/104 (1.9%)	0.84
Any primary site surgery	All stages	3501/3784 (92.6%)	1469/1562 (94.0%)	2032/2222 (91.4%)	0.002
	Stage I	1790/1923 (93.1%)	738/773 (95.5%)	1052/1150 (91.5%)	0.0007
	Stage II	758/783 (96.8%)	286/296 (96.6%)	472/487 (96.9%)	0.817
	Stage III	551/578 (95.3%)	252/263 (95.8%)	299/315 (94.9%)	0.610
	Stage IV	250/330 (75.8%)	133/164 (81.1%)	117/166 (70.5%)	0.024
	Stage unknown	152/170 (89.4%)	60/66 (90.9%)	92/104 (88.5%)	0.612
Any regional nodal surgery	All stages	679/3784 (17.9%)	383/1562 (24.5%)	296/2222 (13.3%)	<0.001
	Stage I	95/1923 (4.9%)	51/773 (6.6%)	44/1150 (3.8%)	0.006
	Stage II	207/783 (26.4%)	115/296 (38.9%)	92/487 (18.9%)	<0.001
	Stage III	253/578 (43.8%)	147/263 (55.9%)	106/315 (33.7%)	<0.001
	Stage IV	113/330 (34.2%)	65/164 (39.6%)	48/166 (28.9%)	0.039
	Stage unknown	11/170 (6.5%)	5/66 (7.6%)	6/104 (5.7%)	0.639

Chemotherapy use was more frequent in the later years analyzed in the overall population: 7.0% in 2004 vs 16.2% in 2015 (OR 2.37; 95% CI 1.15-4.88, p=.0191), as well as in older adults: 3.9% in 2004 vs 11.8% in 2015 (OR 3.22; 95% CI 1.01-10.25, p=.0478). Compared to 2004, primary site surgery use was less frequent in 2010, 2013, 2014 and 2015 in the overall population, and in 2009 in older adults. No significant utilization differences were found in regional lymph node surgery or radiotherapy over time.

## Discussion

In this competing risk analysis, age ≥65 was not significantly associated with a higher incidence of death due to penile cancer. However, OS was lower in older patients with penile cancer, which is expected given the median age for each subgroup and life expectancy relative to the general US population (12). Using competing risk analysis, on univariate analysis older age was associated with a lower incidence of penile cancer death, but on multivariate analysis, this was no longer significant.

Younger patients were more likely to be of Hispanic ethnicity, single, and uninsured. These demographic and socioeconomic factors have been described as predictors of a higher pathologic stage (13). Unmarried status is associated with diagnostic and therapeutic delays, impacting long-term survival (14). We also found that younger patients had a higher prevalence of stage III and IV disease, with 27.3% of younger patients diagnosed with stage III or IV disease, in comparison to 21.7% of older patients. However, when analyzing competitive causes of mortality, we found that penile cancer mortality was higher in younger patients with disease stages II, III and IV when compared to older patients, which may account for the higher prevalence of adverse characteristics in the younger subgroup. Regarding other risk factors, data on HPV status is not available for patients with penile cancer in the SEER database. Other investigations have found a longer CSS in patients with HPV-positive penile cancer (15). However, it is unlikely that this explains the difference in survival we observed: a retrospective study in Brazil reported a similar prevalence of HPV infection between patients older or younger than 60 years (16).

We found lower rates of treatment in older patients in the overall population for chemotherapy and surgery (both primary and regional), and for some stages regarding radiotherapy. Despite this, penile cancer mortality was lower than in younger patients when compared by stage. Penile cancer accounted only for about one fourth of the deaths in older adults, and therefore, taking into account all other causes of mortality, OS was lower in older adults. This suggests that surgical undertreatment of older patients could perhaps be explained due to an increasing comorbidity burden, causing this older population to experience adverse outcomes due to other causes before death due to penile cancer.

We were able to determine that radiotherapy use was higher in older patients with stage I disease, but due to the limitations of the SEER database, we cannot assess if patients who did not receive surgery for stage I disease received alternative local treatments such as brachytherapy, which is feasible in selected older patients (17).

Similarly to other reports (18), chemotherapy use showed a slight increase over time in the overall population and in older adults. Despite this, even though chemotherapy use was not associated with survival, it stands out that chemotherapy use for advanced disease in the whole analyzed population was low (20.8% for stage III and 45.5% for stage IV), and that only 13.7% of older patients with stage III and 37.4% of those with stage IV disease received chemotherapy. The present analysis did not consider factors involved in determining treatment, including patient preference, comorbidities, physician recommendation, or social factors such as distance to the cancer center, which may play a larger role in older adults. The chemotherapy regimens used in penile cancer can have significant toxicity and some oncologists may be reluctant to prescribe these to older patients. In one study, increasing age was found to be a predictor of forgoing palliative treatment for advanced penile cancer (19). In this same study, a Charlson Comorbidity Score of 3 or more, however, was also a significant predictor of choosing to receive palliative treatment. Our findings are similar to other reports of underutilization of guideline-directed surgical (20)and systemic (21) treatment in a general penile cancer population.

Our study has some limitations, including those inherent to its retrospective and observational nature. Emergent molecular prognostic factors, including epidermal growth factor receptor (EGFR), microsatellite instability (MSI), tumor mutation burden (TMB) and programmed death-ligand 1 (PD-L1) expression (22, 23), and detailed information on competing risks, such as comorbidities, which may influence overall survival especially in older adults (24), are not included in the SEER database, and therefore could not be explored. The SEER registries collect data on radiotherapy and chemotherapy as part of the first course of treatment without providing information on regimens or dosing received, or regarding subsequent treatment modalities (25). However, given that very few patients receive second-line or further systemic therapy and the benefit of subsequent systemic treatment is very limited (26), we believe that this issue does not impact the interpretation of our results.

Our results pose an important question: should we have different standards of care for younger and older patients who are diagnosed with penile cancer? According to our findings, less intensive treatments for older adults seem to provide at least similar if not better disease-specific outcomes in comparison to older patients. This shines a light on the importance of obtaining specific information for treating older adults with this neoplasm. In rare diseases such as penile cancer, the standard of care includes enrolling patients in clinical trials when possible. Efforts should be made to design these trials to be representative of real-world practice. With a median age at diagnosis of 68 years, this would require the inclusion of older adults in clinical trials, as encouraged by both the American Society of Clinical Oncology and the United States Food and Drug Administration (27). The few prospective clinical trials in this setting have included mostly younger patients: for example, median age in two phase II studies evaluating chemotherapy regiments included patients with a median age of 57.5 and 64 years (7, 28). We acknowledge the ongoing controversy on how to define someone as an older adult, however, given the low inclusion of older adults in penile cancer trials, and our results providing evidence on different rates of treatment according to age, we propose that 65 years could be an adequate cutoff point to define a patient as an older adult in the context of penile cancer.

Since the incidence of penile cancer is higher in low and middle-income countries, multinational collaborations could alleviate the challenges of creating evidence in this rare disease, which should be a global oncology concern. Ongoing studies will provide additional information on treatment strategies for patients with advanced disease, such as the HERCULES trial (NCT04224740) evaluating pembrolizumab in combination with cisplatin-based chemotherapy, and the InPACT (NCT02305654) study, evaluating neoadjuvant treatment strategies and the role of prophylactic inguinal lymph node dissection.

Recently, the use of geriatric assessment guided interventions has been shown to reduce systemic treatment toxicity in older patients with advanced cancer (29, 30). This becomes of great importance since chemotherapy regimens for advanced penile cancer include platinum-based combinations, commonly associated with significant adverse effects. Perhaps older patients who are found to be frail could receive novel or emerging treatments with a lower rate of toxicity. A recent example in penile cancer includes the tyrosine kinase inhibitor dacomitinib (31). Regarding surgical treatment, geriatric co-management in patients who receive cancer surgery has also been associated with lower 90-day mortality and a higher use of inpatient supportive care services (32).

In conclusion, OS was lower in older adults with penile cancer, but age ≥65 was not associated with a higher incidence of penile cancer death on multivariate analysis. Older patients receive less surgery, chemotherapy and radiotherapy when compared to younger patients. Surgical resection was associated with better penile cancer-specific mortality on multivariate analysis. Competing mortality risks are highly relevant in older adults with penile cancer. Our study is an initial approach to understand the treatment trends and real-world outcomes of older adults with penile cancer. Factors associated with undertreatment of older patients with penile cancer need to be studied, in order to develop treatment strategies tailored for this population.

## Data Availability Statement

Publicly available datasets were analyzed in this study. This data can be found here: https://seer.cancer.gov/data-software/.

## Author Contributions

MB and TF contributed to conception and design of the study. EMo and EMe performed the statistical analysis. MB wrote the first draft of the manuscript. HV-A wrote sections of the manuscript. EK, SK, and PS contributed to a critical review of results and analysis. All authors contributed to interpretation of data, manuscript revision, read, and approved the submitted version.

## Funding

This research used the facilities or services of the Population Health Shared Resources at University of Colorado Cancer Center supported by Cancer Center Support Grant (P30CA046934) from the National Cancer Institute.

## Conflict of Interest

The authors declare that the research was conducted in the absence of any commercial or financial relationships that could be construed as a potential conflict of interest.

The reviewer JW declared a shared committee, NCCN Clinical Practice Guidelines in Oncology (NCCN Guidelines^®^), with the authors PS and TF to the handling Editor.

## Publisher’s Note

All claims expressed in this article are solely those of the authors and do not necessarily represent those of their affiliated organizations, or those of the publisher, the editors and the reviewers. Any product that may be evaluated in this article, or claim that may be made by its manufacturer, is not guaranteed or endorsed by the publisher.
